# Auxin Information Processing; Partners and Interactions beyond the Usual Suspects

**DOI:** 10.3390/ijms18122585

**Published:** 2017-12-01

**Authors:** Thea van den Berg, Kirsten H. ten Tusscher

**Affiliations:** Theoretical Biology, Department of Biology, Utrecht University, 3584 CH Utrecht, The Netherlands; t.vandenberg1@uu.nl

**Keywords:** auxin, information processing, specificity, modeling, regulatory networks

## Abstract

Auxin plays a major role in a variety of processes involved in plant developmental patterning and its adaptation to environmental conditions. Therefore, an important question is how specificity in auxin signalling is achieved, that is, how a single signalling molecule can carry so many different types of information. In recent years, many studies on auxin specificity have been published, unravelling increasingly more details on differential auxin sensitivity, expression domains and downstream partners of the auxin receptors (transport inhibitor response 1 (TIR1) and other auxin signaling F-box proteins (AFB)), transcriptional repressors that are degraded in response to auxin (AUX/IAA) and downstream auxin response factors (ARF) that together constitute the plant’s major auxin response pathways. These data are critical to explain how, in the same cells, different auxin levels may trigger different responses, as well as how in different spatial or temporal contexts similar auxin signals converge to different responses. However, these insights do not yet answer more complex questions regarding auxin specificity. As an example, they leave open the question of how similar sized auxin changes at similar locations result in different responses depending on the duration and spatial extent of the fluctuation in auxin levels. Similarly, it leaves unanswered how, in the case of certain tropisms, small differences in signal strength at both sides of a plant organ are converted into an instructive auxin asymmetry that enables a robust tropic response. Finally, it does not explain how, in certain cases, substantially different auxin levels become translated into similar cellular responses, while in other cases similar auxin levels, even when combined with similar auxin response machinery, may trigger different responses. In this review, we illustrate how considering the regulatory networks and contexts in which auxin signalling takes place helps answer these types of fundamental questions.

## 1. Introduction

The plant hormone auxin plays an important role in a wide range of developmental processes [1] as well as in a wide range of adaptive responses to environmental conditions [2,3]. Well known examples are the auxin-dependent control of cell division and differentiation rates [4], as well as the auxin maxima-dependent patterning of stem cell niches in the main root [5,6] and shoot [7] as well as new lateral organs [8,9,10], and the prepatterning of the plant’s vasculature network [11,12]. Likewise, in most tropisms, the oriented growth of plant organs towards or away from a particular signal is guided by an instructive auxin asymmetry [13,14,15] and remodeling of overall plant root architecture in response to environmental conditions involves changes in auxin distribution patterns [16,17]. This knowledge begs the question as to how a single hormone signal can convey so many different types of information. A large body of research, aimed at answering how specificity in auxin signalling arises, focuses on the different types of auxin receptors (TIR/AFB), Aux/IAA repressors and auxin response factors (ARFs) [18,19] that together form the plant’s major auxin signalling pathway. In Arabidopsis, a total of 6 TIR/AFB auxin receptors [20], 29 AUX/IAA repressors and 23 ARFs have been identified [21], suggesting that part of the specificity in auxin signalling may depend on the specific auxin signalling molecules applied in a specific context. Research in this direction has uncovered differential sensitivity of distinct AUX/IAAs to auxin [22,23] specialised expression domains of different IAAs and ARFs [24,25], as well as specificity differences between ARFs in the binding of auxin response elements in the promotors of downstream target genes [26]. This knowledge enables one to answer certain questions on auxin specificity. As an example, if distinct modules with distinct auxin sensitivities are present within the same tissue, this explains how responses can vary with different levels of auxin. Indeed, the consecutive activation of the IAA28/ARF5,6,7,8,19, the IAA14/ARF7,19 and the IAA12/ARF5 auxin response modules involved in lateral root formation [27] may be related to an increase in auxin levels generated by the currently active module as well as feedbacks between the different modules [28,29]. Similarly, the expression of different auxin response modules with similar auxin sensitivity in different tissues enables us to explain how an identical auxin signal conveys different information in different contexts [25]. Intriguingly, auxin itself appears to often be involved in setting up these auxin response domains [8].

However, the insights on differential auxin sensitivity, expression domains, and downstream targets of different TIR/AFB, AUX/IAA and ARF types are insufficient to answer more complicated questions on auxin specificity. As an example, similar changes in auxin levels, occurring in the same tissues, may need to lead to different responses. To illustrate this, consider a cell at the proximal boundary of the root meristem and the transition zone that experiences an elevation in auxin level. How should this cell interpret this elevation in auxin level?

**Figure 1:**
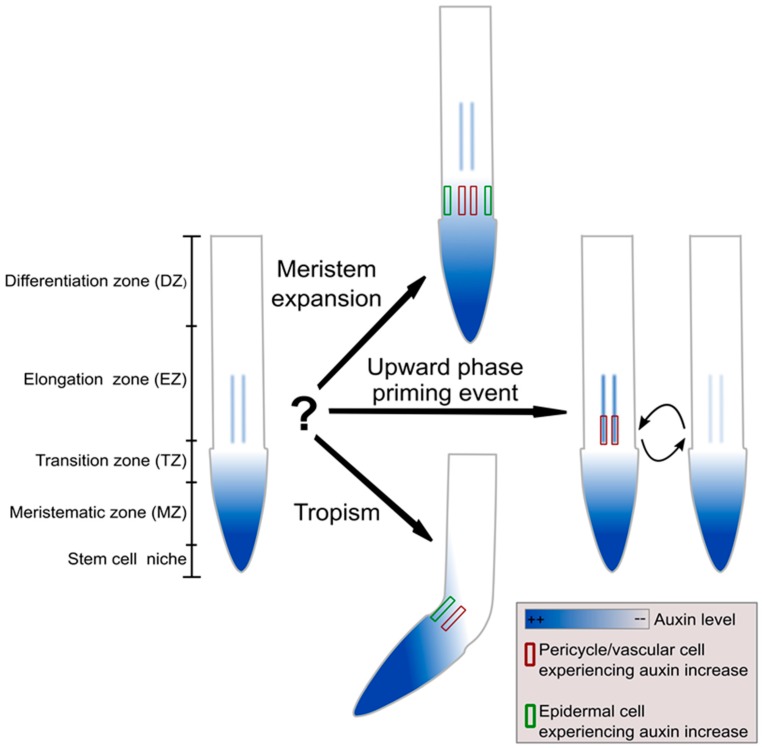
How can an individual plant cell deduce sufficient information from a locally perceived auxin increase? An increase in auxin experienced by an individual cell may reflect a long-term, tissue wide increase in auxin that will result in meristem expansion (**upper graph**, perceived in both epidermal and vascular cells), alternatively it may represent the upward phase of the oscillatory lateral root priming process (**middle graph**, predominantly perceived in vascular cells) and finally it may arise from tropism (**lower graph**, perceived in epidermal and possibly vascular cells). Thus, for an individual epidermal or vascular cell, a perceived auxin increase may arise from at least two of these three different situations, for which a different response is required.

Auxin, combined with PLETHORA (PLT) transcription factors and antagonized by cytokinin, is a major determinant of meristem size [4,30]. Thus, the auxin increase could imply that meristem size is expanding and hence that, rather than loosing meristematic identity and starting to elongate and differentiate, the cell should stay meristematic. Or it could rather imply that the plant organ is undergoing a tropic response and the cell should respond by reducing the elongation rate to support root bending. Alternatively, the elevated auxin level could also inform the cell that it finds itself in the middle of the upward phase of a lateral root priming event. Arguably, gravity responses are likely to be primarily controlled by epidermal auxin asymmetries, while lateral root priming involves auxin oscillations occurring specifically in the protoxylem and overlaying pericycle, and only meristem expansion may be governed by a tissue wide expansion of the auxin gradient. Still, this would require that an epidermal cell can at least distinguish between epidermis dominated asymmetric or rather tissue wide auxin elevations, while pericycle cells should be able to determine whether auxin elevations are pericycle specific or not. Intuitively, for us humans with a mind programmed for pattern recognition, it is clear that the response of the cell critically depends on the duration of the auxin elevation as well as to what extent other cells are experiencing the same or different changes in auxin levels. In the case of a meristem expansion, a persistent root wide change in auxin occurs, whereas in the case of tropism a transient asymmetric change in auxin takes place, while finally in the case of priming a transient more or less symmetric increase in auxin takes place that may be limited to the vasculature (Figure 1). However, it is far less clear how an individual plant cell is to obtain and decode this information on temporal and spatial aspects of auxin dynamics. Indeed, neither differential sensitivities nor differential expression domains of auxin response modules are sufficient to explain this. As another example, differential sensitivities and domains also do not enable us to explain how certain processes can be sensitive for relative rather than absolute changes in auxin levels, eliciting similar responses for widely different auxin levels.

In this review, we argue that to unravel such more complex auxin specificity problems, it is critical to consider the regulatory networks and functional context in which auxin signalling takes place. We will discuss several example studies in which such an approach was successfully applied. The common denominator between and central to the success of these studies is the combination of experiments with computational modelling. Generally speaking, the power of computational models lies in their capability to integrate knowledge obtained on different types of processes, playing out at different spatio-temporal scales, and investigate the types of feedback and emergent properties that these processes together give rise to. In addition, models allow us to vary the processes taken into consideration, their interactions and their conditions, enabling us to narrow down the core processes responsible for a biological property. Specifically, in the context of auxin sensitivity, by integrating auxin controlled processes playing out at different space and timescales, models enable, or even force us to investigate how these processes may functionally co-exist. In addition, they enable us to investigate the consequences of auxin-dependent feedback and auxin concentration ranges.

In the following sections, we discuss how using a modelling approach, studies have found major roles for feedback, differences in time scales, spatial patterning, auxin dependence of auxin transports and players other than the TIR/AFB, AUX/IAA and ARF factors. For example, we illustrate how a recent study demonstrated that auxin can simultaneously and without conflict control both stable developmental zonation and transient tropisms, by applying a division of labour separating the long developmental from the short tropism timescales [30]. We end with the suggestion that plants are likely to have an as-yet uncharacterized machinery that enables them to respond similarly to a change in auxin levels across a wide range of auxin concentrations, similar to the maintained sensitivity of bacterial chemotaxis [31].

## 2. The Auxin–Plethora Division of Labour; A Separation of Timescales

Two hallmarks of plant life are their lifelong continuation of growth and developmental programs and their ability to alter their development in response to environmental conditions. Combining these two characteristics requires dynamic adjustment of developmental programs to a changing environment, yet at the same time stably maintain a meristematic zone and ordered differentiation. Intriguingly, auxin is often involved in controlling both of these seemingly contradictory demands [4,14,15]. As an example, in the plant root, a gradient of auxin controls developmental zonation, with highest auxin levels corresponding to the quiescent center (QC) and surrounding stem cell niche (SCN), and gradually declining levels occurring throughout the rest of the meristem, elongation and differentiation zones [5,6,32,33]. At the same time, auxin affects the rates at which division, elongation and differentiation in these different zones occur [34]. For instance, during gravitropism, when roots grow towards gravity, an asymmetric auxin accumulation leads to a single sided reduction in elongation rates that causes bending of the root towards the gravity vector [35]. In terms of auxin specificity, the question thus is how a transient auxin asymmetry can cause a growth asymmetry yet not perturb the developmental zonation that also appears to be controlled by auxin levels. Assuming that auxin would both alone and directly dictate the developmental stage of a cell and at what rate processes involved in this stage are conducted, implies that an auxin asymmetry involved in tropic responses would perturb the root’s developmental zonation. Therefore, it seems counterintuitive that auxin can control both stable developmental zonation and fast, transient tropisms. It has already been known for a long time that a family of transcription factors called the PLETHORAs (PLT) transcription factors play an important role in plant development [36]. Interestingly, these *PLT* genes are induced by auxin and expressed in a longitudinal gradient resembling the auxin gradient [37]; furthermore, these PLTs are a main determinant for root developmental zonation [36]. To unravel the relative roles of auxin and PLTs and how these may together enable specificity, Mähönen et al. combined experiments with modeling. First, they demonstrated that while auxin directly affects the rates of division, expansion and differentiation, it appears to affect zonation only indirectly [30]. Indeed, ectopic expression experiments demonstrated that PLT levels cell-autonomously control whether cells behave as stem cells, transit amplifying or differentiating cells. In line with this, increasing or reducing native *PLT* expression was shown to expand or reduce meristem size, respectively. Furthermore, they demonstrated that only prolonged exposure to high auxin levels induces *PLT* expression. Incorporating these findings into a multi-scale model of root growth predicts transcription close to the QC where auxin levels are high, thus resulting in a limited PLT protein domain rather than a gradient. The observation that in clonal expression experiments PLT proteins are present slightly outside their transcription domain led the authors to hypothesize that PLT proteins can move through the plasmodesmata that connect the cytoplasm of neighboring cells. Incorporating plasmodesmatal movement into the model demonstrated that a significant expansion of the PLT protein domain beyond its transcriptional domain could indeed arise, provided that PLT protein turnover is sufficiently slow. This can be understood from the fact that movement of PLT proteins through plasmodesmata is slow (order of magnitude of displacement of few cell diameters per 24 h); consequently, proteins will travel only a small distance if they are degraded too fast (half-life of less than 10 h). The authors subsequently experimentally confirmed this predicted importance of PLT protein stability for gradient formation. Finally, by using the model to virtually close the plasmodesmata, it was shown that stable proteins still formed a gradient beyond their transcriptional domain, albeit with a shorter length scale. When cells grow and divide, they become pushed out of the high auxin domain to which *PLT* transcription is limited. However, as a result of high PLT protein stability, protein levels do not immediately drop to very low levels in the absence of de novo transcription and translation PLT levels will drop gradually over time, causing PLT levels to reflect the amount of time or rather the number of cell divisions that have passed since the cell has left the *PLT* transcriptional domain, a process called mitotic segregation [38]. Again, this finding was confirmed experimentally; this indicates that while auxin induces *PLT* transcription, the PLT protein gradient is not a simple readout of the auxin gradient. Instead, *PLT* transcription shows a slow response to high auxin levels, and the resulting spatially limited transcription domain is converted into a protein gradient through the slow processes of mitotic segregation and cell-to-cell movement.

**Figure 2:**
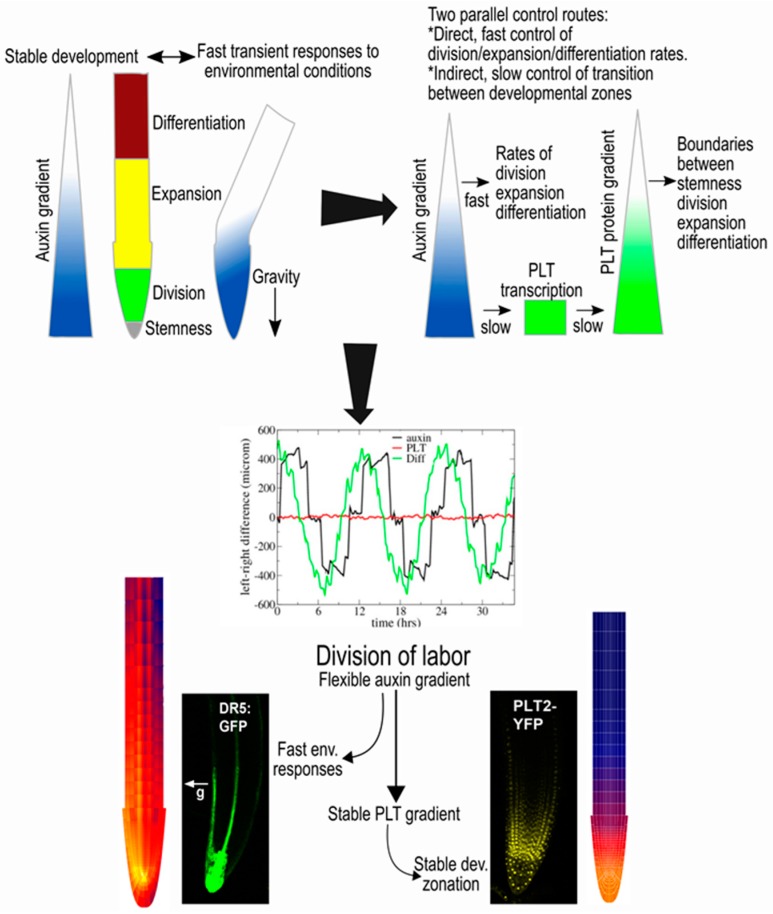
The auxin–PLETHORA (PLT) division of labour [30]. If auxin were to directly control both rates and developmental zones, transient auxin asymmetries occurring during tropisms would perturb developmental zonation (**upper left panel**). PLT gradients result from auxin gradients through slow induction for high auxin levels, slow division and slow cell-to-cell movement (**upper right panel**). The control of rates by auxin and of zones by PLTs enables fast adaptation to tropic cues while maintaining stable PLT-mediated zonation (**middle panel**). Experiments confirmed the model predictions of the division of labor by the partial independence of the auxin and PLT gradients (**lower panel**).

As a consequence, the PLT gradient depends only on the root tip auxin maximum. In addition, the auxin and PLT gradients have different temporal dynamics. Auxin patterns respond directly to changes in expression levels, and the polar orientation of their cellular exporters, the PIN proteins. In contrast, PLT patterns change only in response to prolonged changes in the auxin maximum. Mahonen et al. demonstrated that this partial independence of the auxin and PLT gradients combined with their different timescales is critical for enabling auxin to govern both fast adaptation to environmental conditions and stable developmental zonation [30]. Upon simulated gravitropism, the change in columella PIN polarity leads to the rapid generation of an auxin asymmetry driving gravitropic bending. At the same time, the PLT gradient remains constant, enabling it to maintain a stable boundary between the meristem and elongation zone that is necessary for a temporally ordered, and tissue-wide coordinated progression of cell differentiation (Figure 2).

Auxin can thus fulfill two seemingly conflicting tasks by performing one directly, and the other indirectly using a partner that only partly depends on auxin and has substantially slower dynamics.

## 3. Halotropism as a Case Study of a Graded-Signal Tropism; Auxin Computations in the Reflux Loop

Tropisms form an important aspect of plant adaptation, enabling individual plant organs to grow away or towards particular cues. In all tropisms but hydrotropism [39], the bending of plant organs is orchestrated through an asymmetric auxin pattern that causes asymmetric growth rates [13,40,41]. A major question in tropism research is thus how different environmental stimuli become translated into an instructive auxin asymmetry.

For plant roots, the most studied tropism is gravitropism, the orientation of the root towards the gravity vector. Specialised root tip columella cells containing starch granules, called statoliths, play a major role in gravitropism. Upon re-orientation of the root, the statoliths sediment on the new downward face of the cells [42], causing a change in the pattern of PIN3 and PIN7 proteins from an apolar localisation at all membrane faces to a polar orientation on the now downward face of the columella cells [13,43]. As a consequence, auxin flux is biased to the lower side of the root, causing an elevation of auxin at the lower side and a decrease of auxin at the upper side of the root [44,45]. Importantly, in gravitropism, individual cells, through statolith sedimentation and subsequent PIN repolarization, can sense the direction of and respond directionally to the gravity signal. As a consequence, all columella cells, independent of whether they are on the upward or downward side of the root, coherently polarize their auxin transport towards the lower side, thereby directly generating a clear and robust auxin asymmetry. In plant shoots, the most studied tropism is phototropism, the orientation of the shoot towards light. At the light exposed side, plant cells respond by reducing ATP-binding casette B19 (ABCB19)-mediated downward auxin transport and enhancing PIN3-mediated lateral auxin transport, thus locally accumulating auxin, while at the shaded side no such response occurs. As a consequence, an auxin asymmetry directly arises from the differential reception of and response to the signal at the two sides of the plant hypocotyl [46].

A completely different situation arises in a recently discovered root tropism, halotropism, where roots bend away from elevated salt concentrations [41,47]. Since salt readily diffuses through the medium—experimental agar or soil—roots will generally be exposed to a relatively shallow gradient of salt, rather than experiencing salt only on one side. For example, in the study of Galvan-Ampudia et al. across root salt concentration, differences are in the order of only 5–10% [41]. A similar, graded signal distribution may occur in hydrotropism [48]. As roots experience a salt gradient, this logically implies that cells at the different sides of the root mount a similar response, albeit with cells at the side experiencing more salt a slightly stronger one. Furthermore, it seems unlikely that individual cells are capable of detecting the direction of the salt gradient and responding to it directionally. Thus, the question then is how relatively small differences in salt levels, and hence response strength at the two sides of the root, eventually become translated into a clear-cut overall asymmetry in auxin. To achieve this, cells at different sides of the root should somehow communicate to integrate information from different sides of the root and determine at which side salt concentrations are highest.

**Figure 3:**
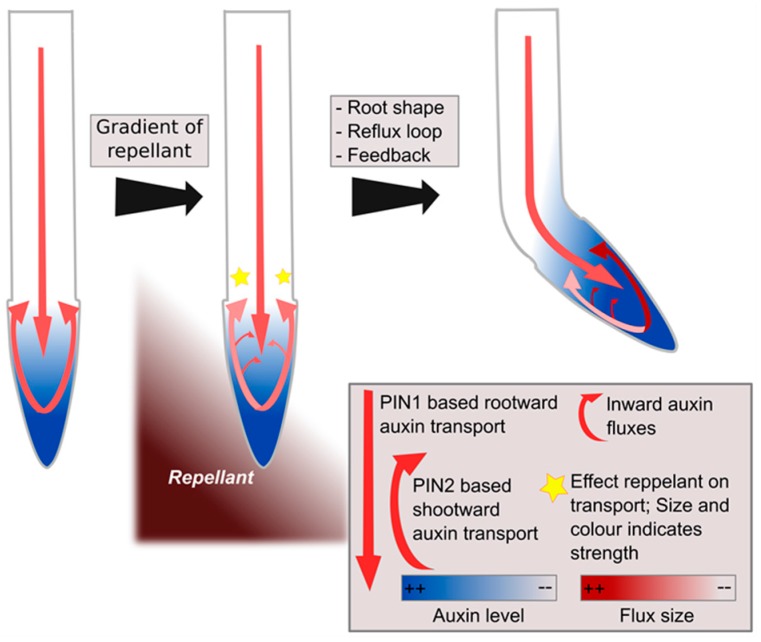
Hypothetical model of left–right sensing in plants during tropisms. A gradient of repellent, for example diffusing NaCl, causes a stress response at both sides of the root, though somewhat stronger at one side than the other, causing modulations in the reflux loop (**middle panel**). Subsequent positive feedback of auxin on its own transporters combined with root tip architecture and reflux loop properties amplify initial differences into a clear instructive auxin asymmetry, enabling bending [49] (**right panel**).

While this specific question has thus far not been addressed, results of a recent study by Van den Berg et al. provide interesting suggestions [49]. In this study, it was shown that the earlier identified asymmetry in the PIN2 auxin exporter [41] is insufficient to fully explain halotropism-induced root bending. The authors used a simulation model to demonstrate that the auxin dependence of the auxin resistant 1 (AUX1) auxin importer and the PIN2 exporter are critical for amplifying the small auxin asymmetry generated by the initial PIN2 asymmetry. Put simply, on the side with highest salt levels, less PIN2 leads to less auxin transport upward on that side, thereby decreasing local auxin levels, which subsequently leads to a further decrease in PIN2 as well as AUX1, etc. As a consequence, increasingly less auxin is transported upward on the more salt-exposed side. The auxin not transported at the side with the highest salt levels is subsequently rerouted to the other side. Initially, only small amounts of auxin will be rerouted; nonetheless, the higher auxin levels, resulting from the rerouting, will amplify AUX1 and PIN2 levels through positive feedback and subsequently increase in auxin rerouting [49]. While the study of Van den Berg et al. does not yet address what generates the initial PIN2 asymmetry, it does point to the important role of auxin feedback on its own transporters as effective amplifiers of initial auxin differences. This opens up the interesting possibility that if the very first response to a graded environmental signal involves qualitatively similar yet quantitatively slightly different changes in auxin transport or signalling, the root tip reflux loop combined with the auxin feedback on auxin transporters may suffice to amplify these initial differences into a full auxin asymmetry (Figure 3).

## 4. Auxin Signalling in Phyllotaxis; Same yet Different

At the shoot apex, the regular formation of new leaf primordia is preceded by the formation of auxin maxima that arise in the vicinity of the shoot meristem where auxin levels are lower. It has long been established that dynamic repolarization of the auxin exporting PIN1 proteins play a major role in the repetitive generation of these auxin maxima [50]. However, for a long time, an open question remained to what extent pattern formation involved only spatio-temporal differences in auxin concentration levels between primordia and central meristem, and to what extent changes in auxin sensitivity and/or downstream targets may also be involved. Ultimately, the impact of auxin signalling on patterning is a product of the local auxin levels and the local auxin sensitivity.

Interestingly, a large-scale expression analysis of the AUX/IAA and ARF factors active in the shoot apical meristem region revealed that similar factors are active across the meristem region albeit with lower levels occurring in the central meristem than in the periphery and young primordia [51]. Thus, neither differential sensitivity nor different domains of different players involved can explain the distinct developmental trajectories of the central region that remains meristematic and the peripheral regions that repetitively produce new leaf organs. This implies that these different developmental fates solely rely on differences in auxin levels and sensing occurring in the different regions. As a consequence, robust patterning requires the persistent generation of significant differences in auxin levels and sensing between these different regions. 

**Figure 4:**
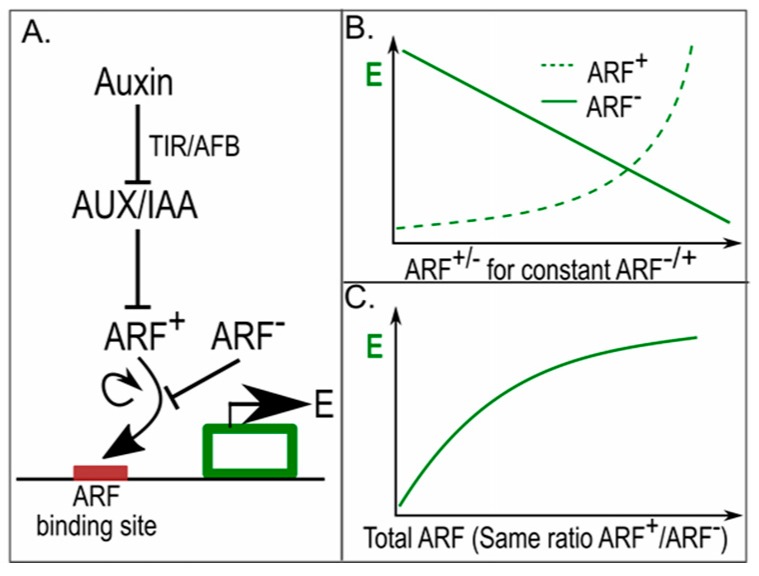
In phyllotaxis, different amounts of ARFs result in differential auxin sensitivity [51]. (**A**) Auxin response network, with auxin de-repressing the AUX/IAA repressed activating ARFs (ARF^+^), activating and repressive ARFs (ARF^−^) competing for the same auxin response elements (ARE) upstream of auxin responsive genes, and gene expression levels (E) cooperatively depending on activating ARFs; Lines ending with arrowheads indicate positive regulatory interactions, lines ending with a horizontal line indicate negative regulatory interactions; (**B**) Gene expression in response to different levels of repressive and activating ARFs, for constant amounts of activating and repressive ARFs respectively. The non-linear response to activating ARFs arises from their cooperative effects on gene expression; (**C**) Gene expression levels in response to different amounts of activating and repressive ARFs for a constant ratio between the activating and repressive ARFs.

Given the observed difference in AUX/IAA and ARF levels between central meristem and periphery, Vernoux et al. applied a modelling approach to investigate the potential significance of these concentration differences for robust auxin-driven patterning. A key aspect of the model is that it incorporates gene expression-activating ARFs that can be repressed by AUX/IAA and derepressed by auxin, but also the less frequently considered autonomously acting repressive ARFs (Figure 4A). Importantly, activating and repressive ARFs compete for the same finite number of binding sites upstream of target genes [52]. Finally, activating ARFs are assumed to cooperatively affect gene expression. As a consequence, while gene expression linearly declines with the number of repressive ARFs it supralinearly increases with the number of activating ARFs (Figure 4B). Using the model, the authors could thus demonstrate that if the number of activating and repressive ARFs increases similarly, downstream gene expression increases (Figure 4C). This enabled them to explain how the lower levels of ARFs and AUX/IAA occurring in the centre of the meristem result in lower auxin sensitivity than the higher levels occurring in the periphery and the primordia. This differential sensitivity was subsequently experimentally confirmed. Furthermore, the spatial correlation between low auxin levels and low auxin sensitivity in the centre and high auxin levels and auxin sensitivity in the periphery was shown to contribute to the robustness of phyllotactic patterning [51]. While not addressed in this study, one can imagine that by making AUX/IAA and ARF levels auxin dependent, sensitivity to auxin becomes correlated with auxin levels.

## 5. Pin Polarity in Gradients; Different yet Same

**Figure 5:**
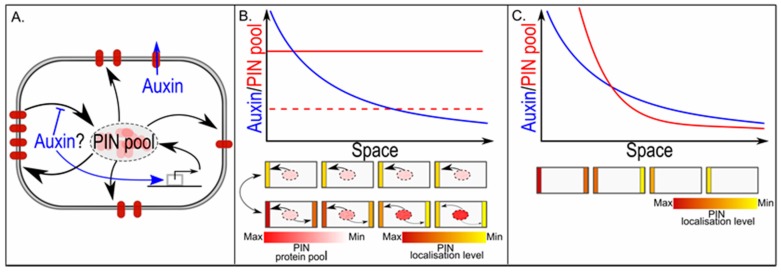
In PIN polarisation, the cellular PIN pool size determines graded versus all-or-none polarisation. (**A**) Gene expression is a major determinant of overall cellular PIN pool size. Individual membrane compartments derive their PINs from this single shared PIN pool, and PINs not deposited on the membrane together constitute the remaining cytoplasmic PIN pool; (**B**) The upper graph shows a hypothetical auxin gradient across a one-dimensional tissue. We assume an up-the-gradient type of feedback on PIN localisation. If the overall cellular PIN pool is small, all-or-none polarisation occurs and all cells show the same polarity pattern with high amounts of PINs on the highest concentration facing membrane and low or absent PINs on the lowest concentration facing membrane, and few PINs left in the cytoplasmic PIN pool (middle figure). If the overall cellular PIN pool is large, PIN levels on each individual membrane depend on the auxin level they experience, resulting in a graded polarity pattern with amount of polarity and amount of PINs on highest and lowest concentration facing membranes increasing along the gradient, and amount of PINs remaining in the cytoplasmic gradient decreasing along the gradient (lower figure); (**C**) The upper graph shows again a hypothetical auxin gradient that now induces a gradient in overall cellular PIN pool sizes. For small PIN pools, all-or-none polarisation occurs; for larger PIN pools, polarisation becomes graded with auxin levels, while for very large PIN pools, apolar PIN patterns arise.

The ability to generate well defined auxin maxima, gradients and paths critically depends on the polar localisation of the auxin exporting PIN proteins [5,53,54]. It is generally assumed that, at least to a certain extent, the polar membrane localisation of PIN proteins depends on auxin (Figure 5A). Unfortunately, how exactly these polar patterns arise remains unclear. Earlier hypotheses on the role of the auxin binding protein 1 (ABP1) protein in sensing auxin levels [55] and the role of auxin-dependent cycling of PIN proteins to and from the membrane [56] in setting up PIN polarity have become heavily disputed due to recent studies [57,58]. Because of this yet incomplete understanding, models for PIN polarity dynamics have mostly been formulated in phenomenological terms. Depending on whether the aim was to explain patterns of shoot phyllotaxis or leaf veination, up-the-gradient or with-the-flux feedbacks of auxin levels or transport on PIN levels have been proposed [59,60,61]. In the former, PIN levels are assumed to increase on membranes oriented to neighbouring cells with high auxin levels; in the latter, PIN levels are assumed to increase in the direction of largest transport flux.

Detailed mathematical analysis of a large range of PIN polarity models showed that, independent of assuming either with-the-flux or up-the-gradient feedback, the type of PIN polarisation patterns arising strongly depended on the size of the cellular PIN pool and the extent to which all PIN proteins are deposited on the membrane [62]. If the amount of PIN proteins in a cell is assumed to be large relative to the amount of PINs that will be localised on the membrane, PIN levels are not limiting. As a consequence, different membranes of the same cell are not competing for PINs, and each membrane can adapt its PIN levels to local auxin or auxin flux levels. Under these conditions, graded PIN polarity patterns arise: if auxin levels or fluxes differ more across a cell, the cellular PIN patterns will polarise more strongly. Thus, along a non-linear auxin gradient, cellular polarisation increases with the steepness of the gradient. Furthermore, as individual membranes respond to their local auxin or flux level, even along a linear auxin gradient resulting in similar polarisation levels, average PIN levels will follow auxin or flux levels. Indeed, if one adds to these models the realistic assumption that membrane compartments can only contain a finite amount of PINs, large unlimiting levels of PINs combined with very high auxin levels generate apolar PIN patterns (Figure 5B). If, in contrast, we assume a finite pool of PIN proteins that for a large part will be positioned on the membrane, PIN proteins become limiting. In this case, the different membrane compartments of the cell compete for PINs; putting more PIN proteins on one membrane automatically means that less PINs will be available for other membranes. Due to positive feedback, more and more PINs will be put on the membrane facing the highest auxin levels or auxin flux levels, and less and less on the other membranes, resulting in full-blown polarisation, independent of the average levels or size of across-cell differences in auxin or auxin flux that the cells were experiencing. This all-or-none polarisation allows cells in different parts of the tissue, experiencing different average flux strengths or concentrations, as well as different across-cell differences in fluxes or concentrations, to build a similar PIN polarity pattern [62] (Figure 5B).

The above demonstrates two things relevant for auxin specificity. First, if the cellular PIN pool to a large extent is localised on the membrane, different auxin levels or fluxes can produce similar PIN polarity patterns. Second, by regulating PIN pool size, similar gradients in auxin levels or fluxes can generate different PIN patterns: all cells polarised similarly, or cellular polarisation changing along the gradient (Figure 5C). In this context, it is noteworthy that auxin, both directly and via regulating the PLT transcription factors, upregulated *PIN* expression levels [53,63].

## 6. Conclusions and Outlook

The plant hormone auxin plays a critical role in a wide range of developmental and adaptive processes. Understanding these processes, at an individual level as well as in relation to one another, requires that we understand how auxin can regulate so many distinct processes. Logically speaking, a single signal, such as auxin, can only convey distinct or even contradicting information by collaborating with other factors. Traditionally, specificity of auxin signalling is considered in terms of differential sensitivity, expression domains or downstream targets of auxin signal transduction pathways. In this article, we argued that other partnerships beyond these usual suspects as well as the context and regulatory networks in which auxin signalling takes place are critical to consider. To support this argument, we demonstrated a series of insights on auxin specificity obtained in recent studies.

While we discussed only a limited number of examples in this review, we expect that the type of partnerships pointed out is more common. For instance, in the last example of PIN polarity, we discussed how different auxin signals can generate similar responses. Because of the finiteness of available PIN proteins, under certain conditions different tissue-level auxin gradients can become translated into similar patterns of PIN polarity [62]. We suspect that something similar should hold for temporal auxin changes; under certain conditions, different changes in auxin levels are capable of eliciting the same response, provided that the temporal direction and relative amount of auxin change, increase or decrease, are similar. As an example, while it is still debated whether lateral root priming involves periodic changes in auxin levels or merely auxin responses [64], one would expect that different environmental conditions or different root developmental ages affect root tip auxin levels as well as baseline and maximum levels of these auxin oscillations. Still, effective lateral root priming should occur under all these conditions. This requires a machinery capable of sensing relative changes in auxin levels rather than absolute auxin levels. Sensitivity to relative changes is well-known from bacterial chemotaxis, in which bacteria are capable of sensing a directional relative difference in chemotactic cue across a wide range of concentrations. The mechanistic basis of this capacity to sense relative differences lies in the presence of a slow timescale negative feedback from average concentration levels to proteins responsible for sensitivity, resulting in a normalisation of sensitivity to average concentration levels [31,65,66]. We expect that for sensing relative auxin changes, the TIR/AFB-AUX/IAA-ARF system may play an important. As the study by Vernoux et al. showed, absolute levels of the AUX/IAAs, ARF repressors and ARF activators may impact the sensitivity for auxin [51]. Extrapolating from their results, one can imagine a system in which AUX/IAA and ARF levels depend on long-term auxin levels, causing increased sensitivity to changes in auxin for persistently high auxin levels. Alternatively, auxin sensitivity could also be modulated by affecting the levels of the more upstream TIR1/AFB factors, as was, for example, shown for bacterial infections [67].

In this review, we solely focused on auxin as a critically important plant hormone; however, many more hormones [68,69], peptides [70], and small RNAs [71] are involved in developmental patterning. Therefore, auxin specificity may also arise from combining similar auxin signals with different types or levels of other signalling molecules. A major factor to consider in this context is cytokinin, for which differential patterns have been clearly established [69]. Nevertheless, this will shift the question to what causes these differential cytokinin patterns. Given the highly intertwined nature of auxin and cytokinin signalling, production, degradation and transport, auxin itself is likely involved in controlling cytokinin patterning [72]. More general, many of the signalling molecules involved in development, either directly or indirectly, have an effect on and are at the same time affected by auxin [73,74,75]. Therefore, a complete and in-depth understanding of auxin specificity will require a further elucidation of the regulatory interactions and mutual patterning of auxin with other hormones and signalling molecules. Similar to the studies described here, we expect a major role for computational modeling in unraveling how such complex signalling and patterning networks endow the auxin signal with its specificity.
